# Chronic Granulomatous Aspergillus Synovitis: a Case Report

**DOI:** 10.4084/MJHID.2013.043

**Published:** 2013-06-06

**Authors:** Aylin Canbolat Ayhan, Korhan Özkan, Cetin Timur, Birol Aktaş, Ayse Bahar Ceyran

**Affiliations:** 1 Istanbul Medeniyet University Goztepe Education and Research Hospital Pediatric Hematology Department, Istanbul, Turkey; 2 Istanbul Medeniyet University Goztepe Education and Research Hospital Department of Orthopaedia and Traumatology, Istanbul, Turkey; 3 Istanbul Medeniyet University Goztepe Education and Research Hospital Pathology Department, Istanbul, Turkey

## Abstract

Aspergillus can cause invasive disease of various organs especially in patients with weakened immune systems. Aspergillus synovitis and arthritis are uncommon types of involvement due to this infection. Approaches to fungal osteoarticular infections are based on only case reports. This paper presents a rare case of chronic granulomatous Aspergillus synovitis in an immunocompromised 5-year old girl who was treated for acute lymphoblastic leukemia.

## Introduction

Aspergillosis is one of the most serious infections for immunosuppressed patients which is caused by a fungus called Aspergillus. Hematological malignancy, prolonged neutropenia and use of broad spectrum antibacterials are some of the risk factors for invasive Aspergillus infections.^1^,^2^ Presence of an underlying neutropenia due to chemotherapy correlates with higher mortality rates in Aspergillus infections. In a study reported by Pagano the incidence of proven or probable invasive aspergillosis among acute leukemia patients was 4.7% and attributable mortality rate (AMR) was 48%.^3^ In another study the incidence was reported as 6.1% and AMR as 38.5%.^4^ In Nosari’s prospective study attributable mortality for aspergillosis was 17.3% and this study supported the idea that mortality rates due to fungal infections have fallen over recent years.^5^ Prompt recognition, application of a correct and diagnostic examination with early initiation of efficacious antifungal agents have diminished the incidence to 12.7% and mortality rates to13% among patients with leukemia.^6^ Herein, we report a case of chronic granulomatous Aspergillus synovitis in a leukemic child which was treated successfully.

## Case Report

**Figure 1, A and B:**
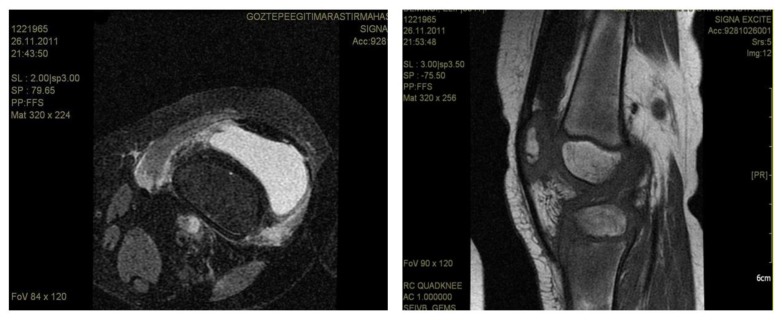
Knee MRI showing synovia inflammation.

A 5-year-old girl who was diagnosed with acute lymphoblastic leukemia and had been receiving maintenance chemotherapy was admitted to hospital with left knee pain and swelling. She had been on chemotherapy for two years. Her maintenance chemotherapy regimen consisted of dexamethasone, mercaptopurine and methotrexate. An initial evaluation revealed the presence of swelling, tenderness and reduced motion of the right knee joint because of pain. She was afebrile and did not have hepatosplenomegaly or lymphadenopathy. Her white blood cell count was 0,6×10^9^/l, absolute neutrophil count was 0,3×10^9^/l. Erythrocyte sedimentation rate was 82 mm/hour. Joint MRI revealed findings consistent with nonspecific severe inflammation of synovia (Figure 1A and B). We stopped her maintenance chemotherapy and performed bone marrow aspiration to see if it was a relapse of leukemia and its bone involvement, but bone marrow was completely in remission. After the consultation with the orthopedia department we decided to perform biopsy from the joint and bone. Biopsy was performed from distal femur and synovia. Surgical debridement of the joint was also performed at the same time.

Bone and synovium specimens examined in the pathology department were consistent with chronic granulomatous Aspergillus synovitis but no speciation was performed. Voriconazole was initiated immediately with a loading dose of 6 mg/kg intravenous every 12 h for two doses and because she was a pediatric patient we decided to follow by 6 mg/kg every 12 h. We also assessed our patient for other organ involvements for invasive aspergillosis but there were no other sites of infection evident on physical examination. A high resolution computed tomography (HRCT) was done to search for a pulmonary source of Aspergillus but it was normal. Hemocultures were negative. Galactomannan test was studied twice a week just after the diagnosis and tests results were negative. We could not find any evidence for a source which could cause a hematogenous or contiguous spread for Aspergillus. Most likely, our patient had isolated Aspergillus synovitis. On the 11 th day of intravenous voriconazole her white blood cell count increased to 2,1×10^9^/l and absolute neutrophil count increased to 1×10^9^/l. During this period she didn’t develop fever in spite of neutropenia On the 15 th day of voriconazole we began to achieve clinical improvement. The response to voriconazole was followed with roentgenograms and also joint MRI. On the 29 th day of treatment her joint MRI was done and it demonstrated remarkable regression in findings of synovitis supporting the good response to voriconazole. At the end of the first month with voriconazole treatment we also began to continue her maintenance chemotherapy. She received intravenous voriconazole for 62 days. A control roentgenogram which was repeated on the 62 nd day supported the clinical improvement. At this time we decided to switch intravenous voriconazole to the oral formulation and we discharged her home on oral voriconazole. She came to hospital once a week for physical examination and complete blood count control. During this period she received the rest of the maintenance therapy and completed her chemotherapy for leukemia. Control roentgenograms were seen approximately once a month and a control MRI was repeated in the 5 th month of treatment with voriconazole. Seven months after the diagnosis of synovitis and initiation of voriconazole follow-up MRI of the knee showed no signs of infection and the treatment was completed. The last MRI did not show any signs of bone loss or destruction in the joint either. Our patient was treated for three months with intravenous voriconazole followed by oral form for the next four months for a total of seven months. We did not put her on prophylaxis because her chemotherapy was completed. At follow-up six months after treatment she remainded well without evidence of infection recurrence.

## Discussion

Aspergillus is a saprophytic mold which is found very common in nature. It’s an opportunistic pathogen.^7^ Aspergillus infections are mostly seen in immunocompromised patients.^8^ Patients with acute leukemia are the most frequent candidates for these infections. Neutropenia due to haematological cancers continues to be an important risk factor for fungal infections.^5^ Renal, lung and heart transplant recipients are also candidates for these infections.^9^,^10^ Joint and bone infections by Aspergillus have also been reported in patients with foreign bodies, after trauma and in wound infections.^11^,^12^ Our patient was predisposed to Aspergillus infection by use of chemotherapy for treatment of acute leukemia. Aspergillus osteomyelitis has been described only as limited case reports in literature and approches to fungal osteoarticular infections are based on these case reports. Because of new developments in diagnosis and early initiation of treatment with new antifungal agents the outcomes of patients have been better in recent years. Aspergillus usually involves the lungs, gastrointestinal tract, brain, skin, sinuses and can causes invasive disease of various organs especially in in immunocompromised patients.^8^ The CNS, cardiovascular system, bones and other tissues may be infected as a result of hematogenous dissemination or direct extension from a contiguous foci of infection.^2^ Osteomyelitis is the fourth most common site of infection for aspergillosis but joint and synovia infections are very rare.^13^ Joint infection can occur as an extension from a focus of Aspergillus osteomyelitis. There are also reported cases of prosthetic joint infections by Aspergillus fumigatus in immunocompetent patients.^14^,^15^ It’s thought that in such cases early and delayed infections may be due to organisms introduced at the time of surgery and late infections may occur as a result of hematogenous spread.^14^,^15^ Spondylodiskitis is one of the most common type of bone involvement reported in literature.^9^,^16^ In children as result of contiguous spread from pulmonary infection the vertebra and ribs are commonly involved.^8^ Interestingly our patient had isolated synovia infection. In our patient the route of infection was unclear. After diagnosis we detected her for presence of any other involved site that could be the primary focus of this joint infection but we didn’t find any other source. We also analyzed her recorded data for the evidence of a prior propable fungal infection at the same site which could be the source of the synotivis by reactivation but we could not find any evidence to support this idea. Galactomannan (GM) test contributes to non-culture-based diagnosis of invasive aspergillosis and serial assessment of galactomannan antigenemia may facilitate therapeutic monitoring.^2^ GM antigenemia is the most important test for obtaining a microbiological criterion for diagnosis. In a study GM antigen was positive in 77% of probable and 53% of proven mould infections.^5^ In our patient galactomannan test was negative. We performed the test twice a week after we obtained the result of the pathologic examination. We did not detect GM antigen before because when complaints about her knee began our patient was neutropenic but afebrile. We did not consider the possibility of fungal infection at the beginning. We think this is very importat too because our case shows that in patients with prolonged neutropenia due to chemotherapy invasive fungal incections must always be in mind even if patients are afebrile. Blood cultures were negative also but it’s well known that even in disseminated infection blood cultures may remain negative. Usually prolonged medical therapy is required for treatment of bone and joint infections.^9^,^13^ The published experience about the treatment of this serious problem is limited. It’s recommended that management of invasive Aspergillosis should be individualized on the basis of clinical criteria such as host defense, underlying disease and site of infection.^2^ Although there are limited data about treatment regimen for Aspergillus osteomyelitis and arthritis surgical intervention combined with medical antifungal treatment is recommended.^2^,^17^ According to Kirby’s review surgical intervention is recommended to reduce fungal load, to remove necrotic material and to increase drug penetration.^13^ Few case reports in literature show that fungal osteomyelitis can develop after arthroscopic reconstruction operations of anterior cruciate ligament (ACL).^18^,^19^ Aggressive bone destruction, osteonecrosis and chondrolysis had been described in these cases.^18^,^19^ Early diagnosis and prompt initiatian of proper antifungal treatment are the major factors which help to reduce the long-term sequele rates and to preserve tha articular cartilage from damage.^19^ Our patient’s last MRI was almost completely normal but we think that we have to follow her very closely for a probable long-term joint sequele. Until recently the choice for invasive aspergillosis was amphotericin B but now it’s well known that its penetration into bone tissues is not good enough. In contrast to this knowledge there are reported cases supporting successful treatment with amphotericin B especially in prosthetic joint infections by A. fumigatus in immunocompetent patients.^14^,^15^ For now voriconazole is the primary choice for treatment of invasive aspergillosis with bone involvement.^16^ Although its penetration into bone tissue has not been well described yet, reported cases support the idea that it’s successful in treating bone infections due to Aspergillus species.^9^,^17^,^20^ Bone infections usually require prolonged antifungal treatment.^16^ According to IDSA guidelines voriconazole is effective for these indications.^2^ In a study satisfactory response at the end of therapy with voriconazole is reported as 55%.^16^ Similarly in our patient vorikonazole was very effective in controlling the infection and it was well tolerated. It’s thought that administration of intravenous or oral voriconazole produces substantial synovial fluid level. There are some reports supporting this idea.^20^,^21^ In Denes’s report the measurements of voriconazole concentrations in the synovial fluid and bone were performed and found that the concentrations were high and similar to agents which have good diffusion to these tissues.^21^ In spite of the subdivision of aspergillus osteomyelitis into subgroups according to involved bones such as vertebral, head, long bones and ribs treatment recommendations don’t show any differences. In Stratov’s study it’s reported that Aspergillus osteomyelitis is to be best treated with surgical debridement of the affected area in conjunction with antifungal therapy.^17^ In our case surgical debridement was performed for eradication of necrotic soft tissue. In Stratov’s study it’s reported that surgery is important especially in cases which AmB is used but if surgery is contraindicated a combination therapy should be prefered.^17^ In guidelines of IDSA surgical resection is recommended for certain conditions such as pulmonary lesions contiguous with the heart or great vessels, invasion of the chest wall, pericardial infection, endocarditis and osteomyelitis. The optimal duration of treatment is not clear yet but it’s known that it usually requires long-term medical therapy. According to guidelines resolution of clinical and radiological findings are very important for determining duration of therapy.^2^ In a study median duration of voriconazole therapy is reported as 83.5 days (range between 4–395 days).^16^ In our case duration of therapy was determined according to improvements in clinical and radiological findings. We followed her with repeated roentgenograms and MRIs. For nonimmunocompromised patients a minimum 6–8 week-treatment is recommended on the other hand for immunocompromised patients the duration is not clear. A long-term treatment throughout the duration of immunosupression is advised.^2^ Our patient was treated for three months with intravenous voriconazole followed by oral form for the next four months for a total of seven months. She had a successful outcome over this time and she was able to receive her maintenance chemotherapy.

In conclusion chronic granulomatous Aspergillus synovitis is very rare but the differential diagnosis of joint pathology should include this infection. In patients with prolonged neutropenia due to chemotherapy invasive fungal incections must always be in mind even if patients are afebrile. In immunocompromised patients Aspergillus can involve any site and leads to very serious infections; because of this clinicians should always have high suspect for fungal infections especially in patients who have weakened immune systems.
